# Operative outcomes, complications, and functional recovery of lateral-position direct anterior approach versus posterolateral approach in hemiarthroplasty: a retrospective cohort study

**DOI:** 10.1186/s12893-026-03742-1

**Published:** 2026-04-21

**Authors:** Daojian Zhang, Zhenning Liu, Liping Pan, Talatibaike Maimaitijuma

**Affiliations:** Department of Orthopedics, Peking University First Hospital, 7 Xishiku Street, Xicheng District, Beijing, 100034 China

**Keywords:** Femoral Neck Fracture, Hemiarthroplasty, Direct Anterior Approach, Posterolateral Approach, Treatment Outcome, Postoperative Complications, Pain Measurement

## Abstract

**Background:**

Hip replacement is a standard treatment for femoral neck fractures, but the optimal surgical approach remains uncertain. This study aimed to compare the clinical outcomes of the lateral-position direct anterior approach (DAA) and the posterolateral approach (PLA) in patients undergoing hemiarthroplasty.

**Materials and methods:**

This retrospective cohort study analyzed clinical data from 56 patients with femoral neck fractures who underwent hemiarthroplasty between February 2019 and September 2022. Patients were classified into a PLA group (*n* = 28) and a DAA group (lateral-position DAA, *n* = 28). Perioperative indices, postoperative pain assessed by the visual analogue scale (VAS), Harris Hip Score, Barthel Index, quality-of-life measures, and early postoperative complications were evaluated.

**Results:**

The DAA group had a shorter operation time (100.54 ± 11.64 min vs. 110.48 ± 22.32 min, *p* = 0.041), smaller incision length (9.31 ± 0.31 cm vs. 12.31 ± 1.21 cm, *p* < 0.001), less intraoperative blood loss (214.95 ± 32.22 mL vs. 268.49 ± 23.53 mL, *p* < 0.001), and shorter hospital stay (10.68 ± 3.31 days vs. 17.49 ± 2.21 days, *p* < 0.001) compared with the PLA group. VAS scores were lower in the DAA group on postoperative days 1, 3, and 7 (all *p* < 0.001). Harris Hip Scores and Barthel Index scores were higher at discharge and at 1 and 3 months postoperatively (all *p* < 0.001). The incidence of early postoperative complications was lower in the DAA group than in the PLA group (7.14% vs. 42.86%, *p* = 0.002), including a reduced rate of hip dislocation (3.57% vs. 25.00%).

**Conclusion:**

Lateral-position DAA using conventional instruments was associated with improved perioperative outcomes, faster early functional recovery, and fewer early complications compared with PLA in this retrospective cohort. These findings suggest that lateral-position DAA may be a feasible alternative approach; however, prospective studies are required to confirm these results.

**Supplementary Information:**

The online version contains supplementary material available at 10.1186/s12893-026-03742-1.

## Introduction

Femoral neck fractures are among the most common fractures in older adults, particularly in women, accounting for approximately 3–4% of all fractures and more than half of hip fractures [1, 2]. The global incidence is expected to continue increasing, with projections exceeding 6 million cases annually by 2050 [3, 4]. These injuries are closely linked to osteoporosis, sarcopenia, and fall risk in the older population [5]. One-year mortality after femoral neck fracture can approach 25%, and many survivors experience persistent functional decline, resulting in substantial societal and healthcare burdens [6, 7]. Surgical management is widely regarded as the standard of care, as nonoperative treatment is associated with markedly increased risks of complications and mortality [8]. The principal goals of surgery are early mobilization, pain control, restoration of hip function, and prevention of immobilization-related complications. Current operative strategies include internal fixation, hemiarthroplasty (artificial femoral head replacement), and total hip arthroplasty. Although internal fixation is less invasive, it is associated with relatively high rates of nonunion (20–35%) and avascular necrosis (5–30%), particularly in displaced fractures and in older patients [9, 10]. Consequently, arthroplasty, especially hemiarthroplasty, is commonly preferred for older or functionally appropriate patients, whereas internal fixation remains an important option for younger individuals [1]. Surgical approach selection is an important determinant of perioperative trauma, early recovery, and postoperative complications after hip arthroplasty. The direct anterior approach (DAA), posterolateral approach (PLA), and direct lateral approach are among the most frequently used techniques [11].

DAA is considered muscle-sparing and has been reported to facilitate earlier functional recovery in some studies; however, it has a learning curve and may be associated with approach-specific complications such as lateral femoral cutaneous nerve irritation. In contrast, PLA is widely adopted because of its familiarity and extensile exposure, yet it has been linked to a higher risk of postoperative dislocation in certain settings due to disruption of posterior soft tissues. Therefore, identifying an approach that balances feasibility, safety, and early functional recovery remains clinically relevant.

Most available comparisons of DAA and PLA have been retrospective and performed with patients in the supine position, often requiring specialized operating tables or instruments. Evidence regarding DAA performed in the lateral recumbent position is comparatively limited, despite the potential advantages of using conventional instruments and maintaining operative familiarity for surgeons accustomed to lateral positioning [12, 13]. Accordingly, this retrospective study compared lateral-position DAA (DAA-lateral) with PLA in patients undergoing hemiarthroplasty for femoral neck fractures. We aimed to evaluate perioperative parameters, postoperative pain, functional recovery, quality of life, and early complications to provide practical and hypothesis-generating evidence for approach selection in this population.

## Materials and methods

### Study ethics

This retrospective study was conducted in accordance with the Declaration of Helsinki (revised 2013) and was approved by the Medical Ethics Council of Peking University First Hospital, Beijing, China (Approval No. ME-TBHP-19–24). The study was conducted and reported in accordance with the STROBE statement for observational studies. Owing to the retrospective design and use of anonymized clinical data, the requirement for written informed consent was waived by the ethics committee. The waiver of consent complied with institutional and national ethical regulations for retrospective studies involving de-identified data.

### General information and study population

**Fig. 1:**
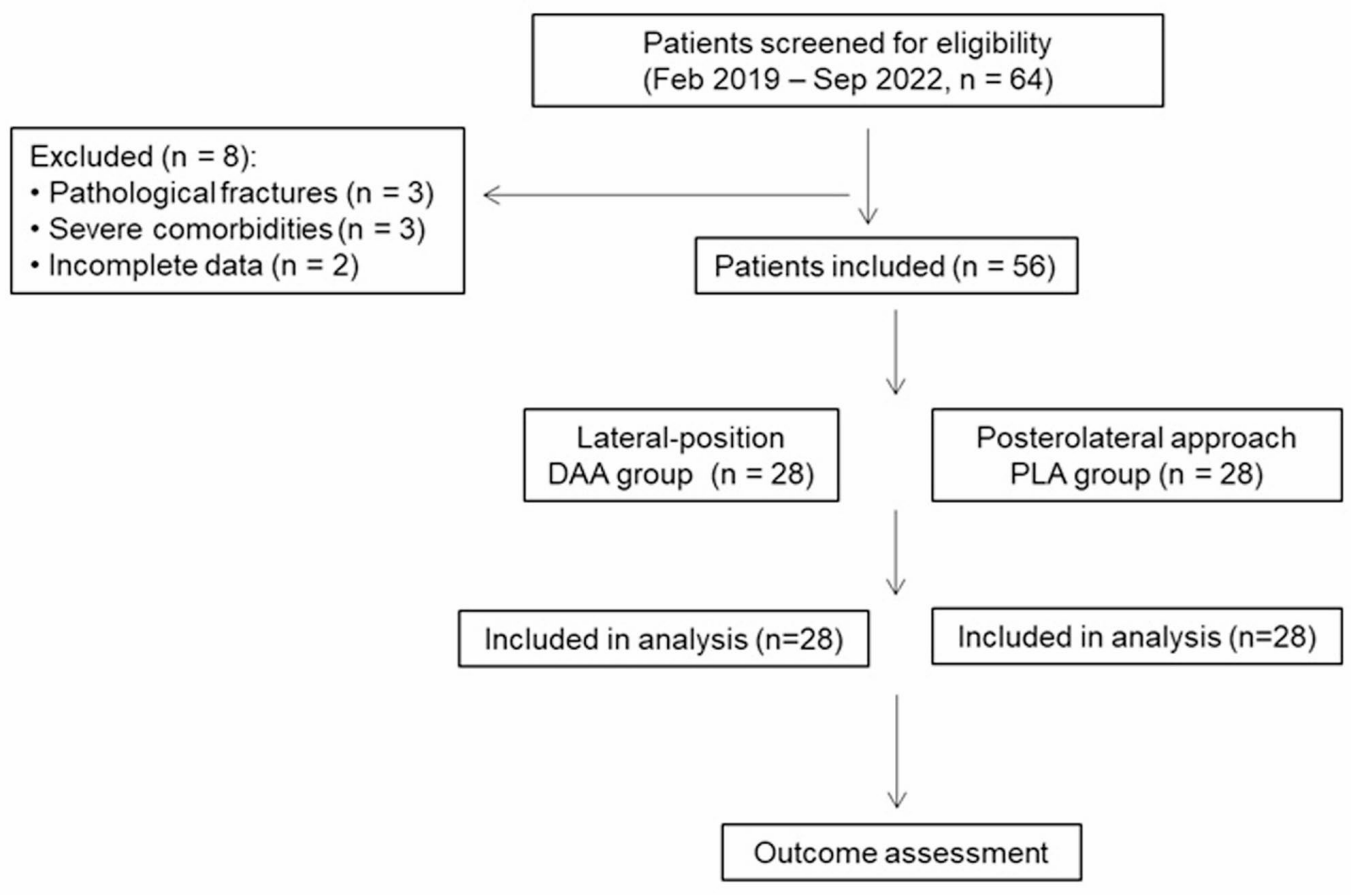
Flowchart of patient selection and group classification. A total of 56 patients with femoral neck fractures who underwent hemiarthroplasty between February 2019 and September 2022 were retrospectively included. Patients were classified into the DAA group, treated with the lateral-position direct anterior approach (*n* = 28), and the PLA group, treated with the posterolateral approach (*n* = 28)

Between February 2019 and September 2022, a total of 64 patients with femoral neck fractures were screened for eligibility at our institution. Eight patients were excluded, including three with pathological fractures, three with severe comorbidities, and two with incomplete clinical data. Consequently, 56 consecutive patients who underwent hemiarthroplasty were retrospectively included and analyzed. Patients were classified according to surgical approach into a PLA group (posterolateral approach, *n* = 28) and a DAA group (lateral-position direct anterior approach, *n* = 28). The choice of surgical approach was based on surgeon preference, familiarity with the technique, and patient-related clinical considerations at the time of treatment rather than random allocation. Therefore, selection bias and residual confounding could not be completely avoided. The patient selection process and study flow are illustrated in Fig. 1. As this was a retrospective study, no prospective sample size calculation was performed, and the final sample size was determined by the number of consecutive eligible patients treated during the study period. However, a post hoc estimation of sample size was performed to assess whether the included cohort was adequate. The required sample size for comparison of two means was estimated using the formula:

$$n=2\times\;\left[\frac{(u_\alpha+u_\beta)\times\sigma}\delta\right]^2$$

where u_α_ is the standard normal deviate corresponding to the significance level (α), u_β_ corresponds to the type II error probability (β), δ represents the expected difference between the two group means, and σ is the pooled standard deviation. Based on previously reported functional outcomes [14], δ = 3.44 and σ = 4.19 were assumed. Under these conditions (α = 0.05, power = 80%), the estimated minimum sample size was approximately 25 patients per group. The final sample size of 28 patients per group in this study therefore met this estimated requirement.

### Inclusion and exclusion criteria

Patients were eligible for inclusion if they had a definitive diagnosis of femoral neck fracture according to established diagnostic criteria [15], met the indications for hemiarthroplasty (including fractures unlikely to heal reliably, comminuted fractures of the lower femoral neck, or poor outcomes following conservative treatment), were aged 40–80 years, were able to ambulate independently and perform activities of daily living prior to injury, had normal cognitive function and communication ability before injury, sustained a low-energy injury such as a slip or fall, and had complete clinical records. Patients were excluded if they had fractures involving other body parts that could interfere with postoperative rehabilitation, a history of old femoral neck fracture, pathological fractures caused by malignancy or osteomyelitis, severe hip osteoarthritis or metabolic bone disease, high-energy trauma (e.g., traffic accidents or falls from height), refusal of surgery or medical contraindications to anesthesia or surgery, incomplete medical records, or severe mental or cognitive disorders.

### Perioperative management

All patients underwent standardized preoperative evaluation, including pelvic radiography and hip joint imaging. Additional investigations, such as chest or brain computed tomography, were performed only when clinically indicated. Routine laboratory assessments included electrocardiography, coagulation profile, liver and renal function tests, and echocardiography when necessary. Preoperative blood pressure and blood glucose were optimized, and anticoagulation management followed institutional protocols. Patients fasted for six hours and abstained from water for two hours before surgery. Prophylactic antibiotics were administered 30 min prior to incision. All procedures were performed under combined spinal–epidural anesthesia. Postoperative rehabilitation protocols, including timing of mobilization and weight-bearing, were standardized across both groups according to institutional guidelines. All procedures were performed by attending orthopedic surgeons with at least 5 years of independent experience in hip arthroplasty and routine familiarity with the assigned surgical approach.

### Surgical techniques

#### Posterolateral approach (PLA)

Patients in the PLA group underwent hemiarthroplasty via the posterolateral approach. A skin incision was made over the lateral aspect of the hip, centered on the greater trochanter, and extended longitudinally along the femoral axis. Standard posterior soft-tissue dissection was performed to expose the hip joint. Following removal of the femoral head and preparation of the femoral canal, the femoral head prosthesis was implanted. Hip stability, leg length, and range of motion were assessed before layered closure, with placement of a drainage tube as required.

### Lateral-position direct anterior approach (DAA)

Patients in the DAA group underwent hemiarthroplasty via the direct anterior approach in the lateral decubitus position. The pelvis was stabilized, and the affected limb was draped free to allow controlled flexion, adduction, extension, and external rotation during femoral exposure. The skin incision was initiated approximately 3 cm distal and 3 cm lateral to the anterior superior iliac spine and extended toward the lateral border of the patella, with a typical length of 8–12 cm. After intermuscular dissection, the internervous and intermuscular interval between the tensor fasciae latae and sartorius was developed, and care was taken to protect the lateral femoral cutaneous nerve. The joint capsule was exposed and incised. The femoral head was removed, and femoral neck osteotomy was performed according to intraoperative assessment and preoperative planning, generally 1.0–1.5 cm above the lesser trochanter with appropriate femoral anteversion. The proximal femur was elevated using standard retractors and limb positioning without the use of a specialized traction table, and the medullary canal was sequentially prepared using broaches. The femoral head prosthesis was implanted. Hip stability, leg length, and joint motion were verified. After thorough irrigation and hemostasis, the capsule and surrounding soft tissues were repaired as appropriate, a drainage tube was placed when indicated, and the incision was closed in layers.

### Outcome measures

Perioperative indices included operation time (minutes), incision length (cm), intraoperative blood loss (mL), length of hospital stay (days), and time to first postoperative ambulation (hours). Intraoperative blood loss was estimated based on suction volume, gauze use, and operative records. Postoperative pain was assessed on postoperative days 1, 3, and 7 using the visual analogue scale (VAS) [16]. Hip function was evaluated using the Harris Hip Score at discharge and at 1 and 3 months after discharge [17]. Quality of life was assessed before surgery and at 3 months postoperatively using a validated 29-item questionnaire covering physiological, psychological, social, and health self-perception domains [18]. Activities of daily living were assessed using the Barthel Index at discharge and at 1 and 3 months postoperatively [19]. Early postoperative complications, defined as events occurring during hospitalization or within 30 days postoperatively, included incision infection, nerve injury, periprosthetic fracture, and hip dislocation, and were diagnosed according to standard clinical criteria. Outcome data were obtained from routine inpatient records and scheduled follow-up documentation. Because of the retrospective design, outcome assessment was based on treating-team records and was not performed by independent blinded assessors, and therefore may be subject to assessment bias.

### Statistical analysis

Statistical analysis was performed using SPSS version 21.0 (IBM Corp., Armonk, NY, USA). Normality and homogeneity of variance were assessed prior to parametric testing. Continuous variables are presented as mean ± standard deviation (SD), and categorical variables as number and percentage [n (%)]. Between-group comparisons were conducted using independent-sample t tests for continuous variables and the χ² test or Fisher’s exact test (when expected cell counts were small) for categorical variables. Repeated-measures analysis of variance was applied to assess group effects, time effects, and group × time interactions for repeated outcomes. Cases with incomplete key clinical data were excluded before analysis, and no imputation of missing data was performed. No subgroup, interaction, or sensitivity analyses were performed. Two-tailed p values < 0.05 were considered statistically significant, with values < 0.001 reported as *p* < 0.001. No multivariable adjustment or matching procedure was performed. Given the relatively small sample size and the limited number of outcome events, especially for individual complications, multivariable modeling was considered likely to produce unstable estimates and increase the risk of overfitting. Accordingly, the present analyses were restricted to unadjusted between-group comparisons and should be interpreted as exploratory and hypothesis-generating. Given the retrospective study design, no randomization or blinding was performed, which is acknowledged as a limitation.

## Results

### Baseline characteristics

In the PLA group, patient age ranged from 43 to 67 years (mean 51.64 ± 3.45 years), including 10 males and 18 females. Body mass index (BMI) ranged from 17.3 to 23.4 kg/m² (mean 21.59 ± 2.21 kg/m²). The time from injury to surgery ranged from 2 to 7 days (mean 3.56 ± 0.42 days), and years of education ranged from 6 to 16 years (mean 10.21 ± 2.10).

In the DAA group, patient age ranged from 44 to 69 years (mean 52.10 ± 3.58 years), including 11 males and 17 females. BMI ranged from 17.5 to 23.7 kg/m² (mean 21.42 ± 2.65 kg/m²). The time from injury to surgery ranged from 2 to 6 days (mean 3.52 ± 0.44 days), and years of education ranged from 6 to 16 years (mean 10.17 ± 2.07). Baseline demographic and clinical characteristics were comparable between the two groups, with no statistically significant differences observed (all *p* > 0.05) (Supplementary Table S1).

### Comparison of perioperative indices

Perioperative indices differed significantly between the two groups. Compared with the DAA group, the PLA group had a longer operation time (110.48 ± 22.32 vs. 100.54 ± 11.64 min, *p* = 0.041), larger incision length (12.31 ± 1.21 vs. 9.31 ± 0.31 cm, *p* < 0.001), greater intraoperative blood loss (268.49 ± 23.53 vs. 214.95 ± 32.22 mL, *p* < 0.001), longer hospital stay (17.49 ± 2.21 vs. 10.68 ± 3.31 days, *p* < 0.001), and longer time to first postoperative ambulation (34.19 ± 2.12 vs. 30.55 ± 2.14 h, *p* < 0.001) (Table 1).

**Table 1:** Perioperative indices (mean ± SD) in lateral DAA and PLA groups

Group	*N*	Operation time (min)	Incision length (cm)	Intraoperative blood loss (mL)	Length of hospital stay (days)	Time to first ambulation (h)
PLA group	28	110.48 ± 22.32	12.31 ± 1.21	268.49 ± 23.53	17.49 ± 2.21	34.19 ± 2.12
DAA group	28	100.54 ± 11.64	9.31 ± 0.31	214.95 ± 32.22	10.68 ± 3.31	30.55 ± 2.14
t		2.089	12.708	7.100	9.054	6.394
p		0.041	< 0.001	< 0.001	< 0.001	< 0.001

### Comparison of VAS scores

Postoperative pain scores decreased over time in both groups. On postoperative day 1, the DAA group had lower VAS scores than the PLA group (4.49 ± 0.89 vs. 5.43 ± 0.94, *p* = 0.0003). On postoperative day 3, VAS scores remained lower in the DAA group (3.41 ± 0.53 vs. 4.58 ± 0.21, *p* < 0.001). By postoperative day 7, the difference persisted (1.28 ± 0.21 vs. 2.39 ± 0.22, *p* < 0.001) (Table 2). Repeated-measures ANOVA demonstrated significant group, time, and group × time interaction effects (all *p* < 0.001).

**Table 2:** VAS pain scores (mean ± SD) on postoperative days 1, 3, and 7

Group	*N*	Day 1	Day 3	Day 7
PLA group	28	5.43 ± 0.94	4.58 ± 0.21	2.39 ± 0.22
DAA group	28	4.49 ± 0.89	3.41 ± 0.53	1.28 ± 0.21
t		3.842	10.859	19.312
p		0.0003	< 0.001	< 0.001

Repeated-measures ANOVA showed a significant group effect (F = 12.423, *p* < 0.001), time effect (F = 18.533, *p*< 0.001), and group × time interaction (F = 15.420, *p*< 0.001)

### Comparison of Harris Hip Score

Harris Hip Scores were higher in the DAA group at all evaluated time points. At discharge, scores were 65.67 ± 3.53 in the DAA group and 60.66 ± 3.66 in the PLA group (*p* < 0.001). At 1 month after discharge, scores were 78.12 ± 1.54 versus 68.86 ± 2.53 (*p* < 0.001). At 3 months, the DAA group continued to show higher scores (86.64 ± 3.34 vs. 75.14 ± 4.55, *p* < 0.001) (Table 3). Repeated-measures ANOVA demonstrated significant group, time, and interaction effects (all *p* < 0.001).

**Table 3:** Harris Hip Scores (mean ± SD) at discharge, 1 month, and 3 months

Group	*N*	Discharge	1 month	3 months
PLA group	28	60.66 ± 3.66	68.86 ± 2.53	75.14 ± 4.55
DAA group	28	65.67 ± 3.53	78.12 ± 1.54	86.64 ± 3.34
t		5.213	16.543	10.781
p		< 0.001	< 0.001	< 0.001

Repeated-measures ANOVA showed a significant group effect (F = 23.112, *p* < 0.001), time effect (F = 16.434, *p* < 0.001), and group × time interaction (F = 23.354, *p*< 0.001)

### Comparison of quality-of-life scores

Before surgery, there were no significant differences between the two groups in physiological, psychological, social, or health self-perception scores (all *p* > 0.05). At 3 months postoperatively, the DAA group demonstrated lower scores (indicating better quality of life) across all domains, including physiological (11.53 ± 2.12 vs. 13.67 ± 2.52, *p* = 0.001), psychological (12.23 ± 1.66 vs. 14.23 ± 4.64, *p* = 0.036), social (12.54 ± 3.75 vs. 16.12 ± 2.94, *p* = 0.0002), and health self-perception (10.85 ± 2.22 vs. 13.92 ± 1.55, *p* < 0.001) (Table 4). Within-group comparisons also showed significant improvements from baseline in both groups (*p* < 0.05), with greater changes observed in the DAA group.

**Table 4:** Quality-of-life scores (mean ± SD) before surgery and at 3 months

Group	*N*	Physiological	Psychological	Social	Health self-perception
PLA (Before)	28	15.19 ± 4.87	16.44 ± 3.65	18.19 ± 3.66	15.65 ± 3.21
PLA (After)	28	13.67 ± 2.52ᵃ	14.23 ± 4.64ᵃ	16.12 ± 2.94ᵃ	13.92 ± 1.55ᵃ
DAA (Before)	28	15.53 ± 4.52	16.48 ± 3.12	18.64 ± 3.92	15.75 ± 3.53
DAA (After)	28	11.53 ± 2.12ᵇ	12.23 ± 1.66ᵇ	12.54 ± 3.75ᵇ	10.85 ± 2.22ᵇ

Values are presented as mean ± SD

ᵃ*p* < 0.05 vs. before surgery within the PLA group

ᵇ*p*< 0.05 vs. before surgery within the DAA group

### Comparison of Barthel Index scores

Barthel Index scores were higher in the DAA group at all time points. At discharge, scores were 56.88 ± 4.32 in the DAA group and 44.54 ± 3.12 in the PLA group (*p* < 0.001). At 1 month after discharge, scores were 65.76 ± 4.21 versus 54.42 ± 5.64 (*p* < 0.001). At 3 months, the difference remained significant (84.23 ± 5.76 vs. 78.66 ± 4.54, *p* < 0.001) (Table 5). Repeated-measures ANOVA demonstrated significant group, time, and interaction effects (all *p* < 0.001).

**Table 5:** Barthel Index scores (mean ± SD) at discharge, 1 month, and 3 months

Group	*N*	Discharge	1 month	3 months
PLA group	28	44.54 ± 3.12	54.42 ± 5.64	78.66 ± 4.54
DAA group	28	56.88 ± 4.32	65.76 ± 4.21	84.23 ± 5.76
t		12.253	8.525	4.018
p		< 0.001	< 0.001	< 0.001

Repeated-measures ANOVA showed a significant group effect (F = 26.594, *p* < 0.001), time effect (F = 26.455, *p* < 0.001), and group × time interaction (F = 33.112, *p* < 0.001)

### Comparison of early postoperative complications

The overall incidence of early postoperative complications was lower in the DAA group than in the PLA group (7.14% vs. 42.86%, *p* = 0.002, Fisher’s exact test). In the PLA group, hip dislocation was the most common complication (25.00% vs. 3.57%). Other complications included incision infection (7.14% vs. 3.57%), nerve injury (3.57% vs. 0.00%), and periprosthetic fracture (7.14% vs. 0.00%) (Table 6).

**Table 6:** Early postoperative complications [n (%)]

Group	*N*	Incision infection	Nerve injury	Periprosthetic fracture	Dislocation	Total
PLA group	28	2 (7.14%)	1 (3.57%)	2 (7.14%)	7 (25.00%)	12 (42.86%)
DAA group	28	1 (3.57%)	0 (0.00%)	0 (0.00%)	1 (3.57%)	2 (7.14%)

Values are presented as n (%). Fisher’s exact test was used for between-group comparison due to small expected cell counts

## Discussion

Femoral neck fractures represent a major clinical challenge in the older population, and surgical intervention remains the cornerstone of management. The choice of surgical approach in hip arthroplasty is known to influence perioperative trauma, early functional recovery, and postoperative complication rates [20, 21]. In this retrospective study, we compared the lateral-position direct anterior approach (DAA) with the posterolateral approach (PLA) in patients undergoing hemiarthroplasty. Our results suggest that lateral-position DAA was associated with more favorable perioperative outcomes, improved early functional recovery, and a lower incidence of early postoperative complications. Specifically, patients treated with lateral-position DAA experienced shorter operative times, smaller incision lengths, reduced intraoperative blood loss, earlier postoperative ambulation, and shorter hospital stays compared with those treated using PLA. These findings are consistent with previous reports indicating that DAA, as a muscle-sparing approach, may minimize soft-tissue disruption and may facilitate faster postoperative recovery [13, 22, 23]. Preservation of posterior capsular structures and avoidance of detachment of the short external rotators may partly explain the observed reductions in blood loss and faster mobilization.

Functional outcomes assessed using the Harris Hip Score and Barthel Index were higher in the lateral DAA group throughout the early postoperative period. In addition, patients undergoing lateral-position DAA demonstrated better quality-of-life scores at 3 months postoperatively across physiological, psychological, social, and health self-perception domains. These findings indicate a potential association between reduced surgical trauma and earlier recovery of independence and daily functioning, although causal inference cannot be established due to the study design. Similar functional advantages of DAA over posterior approaches have been reported in both observational studies and meta-analyses [13, 23, 24].

Importantly, the incidence of early postoperative complications, particularly hip dislocation, was lower in the lateral DAA group. Hip dislocation remains one of the most concerning complications after hemiarthroplasty, often leading to prolonged hospitalization, reoperation, and reduced patient satisfaction. The higher dislocation rate observed in the PLA group is consistent with prior evidence attributing this risk to posterior capsular disruption and injury to the short external rotator muscles [2, 25–27]. By contrast, the lateral-position DAA preserves posterior soft-tissue structures, which may contribute to enhanced joint stability in the early postoperative period.

Recent evidence from a network meta-analysis [24] comparing minimally invasive approaches, including DAA, SuperPATH, and conventional approaches, has also suggested potential advantages of anterior-based approaches in reducing dislocation risk and improving early functional outcomes. Our findings are broadly consistent with this emerging evidence, although direct comparisons should be interpreted with caution due to differences in study design and patient populations. Most previous studies evaluating DAA have been performed with patients in the supine position and frequently relied on specialized operating tables or instruments [28–30]. In contrast, our study demonstrates that DAA performed in the lateral decubitus position using conventional surgical equipment can achieve comparable outcomes within the context of this retrospective cohort. This finding has practical implications, particularly for institutions with limited access to specialized operating systems, and supports the broader applicability of lateral-position DAA in routine clinical practice.

Several limitations of this study should be acknowledged. First, this was a retrospective non-randomized study, and the surgical approach was selected according to surgeon preference and clinical considerations rather than allocation by protocol. This creates an inherent risk of selection bias and confounding. Although baseline variables appeared comparable between groups, unmeasured confounders and selection-related differences may still have influenced the results. Second, no multivariable adjustment was performed. Although adjusted analyses can strengthen observational comparisons, the relatively small sample size and limited number of outcome events in this study would have increased the risk of model overfitting and unstable estimates. Therefore, the findings should be interpreted as exploratory and hypothesis-generating. Third, the relatively small sample size and single-center design may limit external validity and generalizability to other institutions and surgeons. Fourth, outcome assessors were not blinded, which may have introduced assessment bias. In addition, although postoperative rehabilitation protocols were standardized, variations in surgeon experience may still have influenced outcomes. Finally, follow-up in this study was limited primarily to early postoperative outcomes, and long-term endpoints such as implant survival, late complications, reoperation, and sustained functional recovery were not evaluated. Future studies should focus on multicenter, prospective randomized controlled trials with larger sample sizes to confirm these findings. Longer follow-up is essential to further validate the durability and long-term clinical benefits of the observed outcomes.

## Conclusion

In conclusion, lateral-position direct anterior approach hemiarthroplasty was associated with improved perioperative outcomes, faster early functional recovery, and a lower incidence of early postoperative complications, particularly hip dislocation, compared with the posterolateral approach in this retrospective cohort. Because of the retrospective non-randomized design, small single-center sample, absence of adjusted analyses, and limited follow-up, these findings should be interpreted cautiously. Nevertheless, the present study provides preliminary hypothesis-generating evidence that lateral-position DAA using conventional instruments may be a feasible and potentially advantageous alternative for hemiarthroplasty in patients with femoral neck fractures. Further prospective multicenter studies with longer follow-up are warranted.

## Supplementary Information

Supplementary Material 1.

Supplementary Material 2.

## Data Availability

The datasets used and/or analysed during the current study are available from the corresponding author on reasonable request.

## Abbreviations

DAA: Direct Anterior Approach
PLA: Posterolateral Approach
VAS: Visual Analog Scale
ADL: Activities of Daily Living
BMI: Body Mass Index
SD: Standard Deviation
SPSS: Statistical Package for the Social Sciences
MCID: Minimal Clinically Important Difference
