# Supplementary material for: Operative outcomes, complications, and functional recovery of lateral-position direct anterior approach versus posterolateral approach in hemiarthroplasty: a retrospective cohort study
Source: BMC Surg. 2026 Apr 21;26:396. doi: 10.1186/s12893-026-03742-1 (PMC13251276; doi:10.1186/s12893-026-03742-1)
Supplement: Supplementary file 1 — Supplementary Material 1. [file 12893_2026_3742_MOESM1_ESM.docx]

**Supplementary Table S1.** Baseline characteristics of patients in the lateral-position DAA and PLA groups

| Characteristic | Control group (PLA) (n = 28) | Study group (DAA) (n = 28) | *p* value |
| --- | --- | --- | --- |
| Age (years) | 51.64 ± 3.45 (43–67) | 52.10 ± 3.58 (44–69) | > 0.05 |
| Sex (male/female), n | 10 / 18 | 11 / 17 | > 0.05 |
| Body mass index (kg/m²) | 21.59 ± 2.21 (17.3–23.4) | 21.42 ± 2.65 (17.5–23.7) | > 0.05 |
| Time from injury to surgery (days) | 3.56 ± 0.42 (2–7) | 3.52 ± 0.44 (2–6) | > 0.05 |
| Years of education (years) | 10.21 ± 2.10 (6–16) | 10.17 ± 2.07 (6–16) | > 0.05 |
