# Supplementary material for: Operative outcomes, complications, and functional recovery of lateral-position direct anterior approach versus posterolateral approach in hemiarthroplasty: a retrospective cohort study
Source: BMC Surg. 2026 Apr 21;26:396. doi: 10.1186/s12893-026-03742-1 (PMC13251276; doi:10.1186/s12893-026-03742-1)
Supplement: Supplementary file 2 — Supplementary Material 2. [file 12893_2026_3742_MOESM2_ESM.pdf]

STROBE Checklist

Section	Item No	Recommendation	Page No
**Title and abstract**	1(a)	Study design indicated (retrospective cohort)	**1**
	1(b)	Informative abstract provided	**1**
**Introduction**	2	Background and rationale explained	**2–3**
	3	Objectives clearly stated	**3**
**Methods**	4	Study design presented early	**3**
	5	Setting, location, and dates described	**3–4**
	6(a)	Eligibility criteria and participant selection described	**4–5**
	6(b)	Not applicable (no matching)	**NA**
	7	Variables (outcomes, exposures, etc.) defined	**5–6**
	8*	Data sources and measurement methods described	**5–6**
	9	Efforts to address bias described	**6**
	10	Study size explanation provided	**6**
	11	Handling of quantitative variables explained	**6–7**
	12(a)	Statistical methods described	**7**
	12(b)	Subgroup/interaction analyses (not performed)	**7**
	12(c)	Missing data handling described	**7**
	12(d)	Loss to follow-up (not applicable/limited)	**7**
	12(e)	Sensitivity analysis (not performed)	**7**
**Results**	13(a)	Participant numbers at each stage	**8**
	13(b)	Reasons for exclusion described	**8**
	13(c)	Flow diagram (if included)	**8 / Fig. 1 (if added)**
	14(a)	Baseline characteristics reported	**8–9 (Table 1)**
	14(b)	Missing data reported	**8–9**
	14(c)	Follow-up duration summarized	**9**
	15	Outcome data reported	**9–11**
**Main results**	16(a)	Effect estimates and p-values reported	**9–11**
	16(b)	Category boundaries (if applicable)	**6–7**
	16(c)	Absolute risk (not applicable)	**NA**
**Other analyses**	17	Subgroup/sensitivity analyses (not performed)	**7 / 11**
**Discussion**	18	Key results summarized	**12–13**
	19	Limitations discussed	**13–14**
	20	Interpretation provided	**12–14**
	21	Generalisability discussed	**14**
**Other information**	22	Funding statement provided	**15**
